# Shelf life effects on the bond strength and microhardness of self-adhesive resin cements

**DOI:** 10.4317/jced.61362

**Published:** 2024-05-01

**Authors:** Nayra-Kawana Turini, Sandrine-Bittencourt Berger, Murilo-Baena Lopes, Ricardo-Petri Silva, Delise-Pellizzaro Contreras, Terezinha-de Jesus Carvalho-Ferreira, Alcides Gonini-Júnior, Ricardo-Danil Guiraldo

**Affiliations:** 1 Department of Restorative Dentistry, School of Dentistry, University Anhanguera –UNOPAR, Londrina, Paraná, Brazil; 2 Department of Restorative Dentistry, School of Dentistry, University Anhanguera – Uniderp, Campo Grande, Mato Grosso do Sul, Brasil; 3 Department of Electrical Engineering, School of Electrical Engineering, State University of Londrina, Londrina, Paraná, Brazil; 4 Department of Restorative Dentistry, School of Dentistry, State University of Londrina, Londrina, Paraná, Brazil

## Abstract

**Background:**

Among the main advantages of self-adhesive resin cements comprise good aesthetics, strong restoration-tooth bond and biocompatibility. However, some disadvantages, such as high viscosity level, color limitation and short shelf life should be mentioned. Thus, the aim of the current study was to assess bond strength between fiberglass post and root dentin in teeth subjected to self-adhesive resin cements with expired shelf life and hardness.

**Material and Methods:**

Sixty (60) single-rooted human teeth were sectioned and divided into 2 groups of different cements: U200 3M and MaxCem Elite Kerr. Each group was divided into 3 subgroups, based on self-adhesive resin cements’ shelf life, namely: Within the use-time recommended by the manufacturer or no expiration date; 6 months after opening the aluminum blister; 12 months after opening the aluminum blister. Bond strength was measured through push-out test conducted in universal testing machine; fracture pattern was analyzed, and microhardness was investigated through Knoop test, based on hardness readings. Data were subjected to Shapiro-Wilk normality test; nonparametric test was applied to hardness data, whereas parametric test was applied to bond strength data. Hardness data were subjected to Kruskal-Wallis test, whereas bond strength data were subjected to analysis of variance, which was followed by Tukey test; both tests were conducted at 5% significance level (α = 0.05).

**Results:**

There was no statistically significant difference in knoop hardness values recorded for the material / time / root thirds combination (p=0.483). There was no statistically significant difference in bond strength values recorded for the Material / Time / Thirds combination (p=0.237).

**Conclusions:**

It was possible concluding that shelf life did not influence material’s hardness and bond strength.

** Key words:**Dental cements, Resin Cements, Shelf Life of Products.

## Introduction

The emergence of self-adhesive resin cements helped simplifying dental restoration techniques, minimizing operative time and reducing the likelihood of procedural failures, due to their ability to adhere to the dentin in a single step, without the need of performing any previous treatment or surface conditioning (1-3).

The bonding mechanism of self-adhesive resin cements is based on the action of acidic monomers, which act on the dental substrate; such an action simultaneously causes demineralization and cementing agent infiltration into enamel and dentin. This process results in adhesion due to micromechanical retention and chemical interaction between monomers and hydroxyapatite (4).

The main advantages of self-adhesive resin cements comprise lower infiltration and marginal staining level, good aesthetics, lower postoperative sensitivity, strong restoration-tooth bond, lower susceptibility to moisture, biocompatibility and dimensional stability. However, some disadvantages, such as high viscosity level, color limitation and short shelf life should be mentioned (5). Studies carried out with composite resins have shown that shelf life expired after 6 months, and 1 year, did not affect the mechanical properties of the investigated composites (6,7). Accordingly, another study has shown that composite resins expired after 15 months did not affect the mechanical properties of the tested composites (8).

The change in cement properties may be related to the chemical composition of the material itself in terms of filler content or activation mode, or dependent on external factors, e.g., adsorption media or aging (9). Moreover, manufacturers recommend that, after the silver casing is opened, self-adhesive resin cements must be used within 6 months or at most 18 months after the manufacturing date. However, the cement is oftentimes stored for several months before purchase. In addition, the packaging holds sufficient amounts of it to be used in several cementation procedures. Thus, dental clinics with low or medium patient flow are not capable of using all the whole resin cement content in the packaging within the recommended time, and it can lead to material surplus and waste.

The literature lacks studies focused on investigating how long self-adhesive resin cements kept under ideal storage conditions, at low temperature and protected from exposure to light can be used for after their expiration date, without compromising their physical properties and bond strength with the remaining tooth. The aim of the present study was to assess bond strength between fiberglass post and root dentin in teeth subjected to self-adhesive resin cements with expired shelf life (6 months and 1 year) and hardness.

## Material and Methods

The research project was submitted to, and approved by, the Research Ethics Committee of Universidade Norte do Paraná (CAAE 43526521.0.0000.0108), under opinion n. 4.786.830.

-Sample size calculation

Sample size calculation was performed using Minitab 16 software for Windows 8 (Minitab, State College, PA, USA) and selection the ANOVA test, all date were based on the results of a previous study (10). Which recorded bond strength of human premolars, which had a standard deviation of 4.9 by taking into account the minimum detecTable difference of 8.8 on average; an alpha-type error of 0.05, and a beta power of 0.8 were stipulated. Thus, the minimum sampling estimation was considering 9 samples.

-Sample collection

Sixty human teeth were obtained through donation; inclusion criteria comprised single-rooted teeth, without caries or endodontic treatment, with at least 14-mm root length (11), extracted for therapeutic reasons from patients in the age group 18-50 years.

-Sample preparation

Periodontium was cleaned with the aid of McCall curettes 13-14; teeth were stored in separate, frozen in saline solution and kept in freezer until all samples necessary for the experiment were collected.

Teeth were thawed and cut close to the cementoenamel junction, perpendicularly to its long axis, with the aid of cutting machine (Isomet 1000, Buehler Ltd, Lake Bluff, IL, USA) equipped with diamond disk (Extec 12205, Extec Corp. Enfield, USA), which was used under water cooling. Thus, roots, at least 14 mm in length, were obtained; they had their pulp remnants removed based on using extirpate nerves Dentsply Maillefer, Ballaigues, Switzerland).

The apical stop was performed 1mm from the apical foramen, based on using K-files ranging from 15 to 40 (Dentsply Maillefer); dental canals were irrigated with 2 mL of 2.5% NaClO (Phloraceae, Londrina, PR, Brazil) between instrumentations, at each file change. After the instrumentation procedure was over, the conduit was irrigated with 2 mL of 17% EDTA (Phloraceae, Londrina, PR, Brazil); this solution was allowed to remain in the conduit for 5 minutes. Next, dental canals were washed and irrigated with 5 mL of saline solution (Eurofarma Laboratories, São Paulo, SP, Brazil). All roots were filled with AH plus jet endodontic cement (Dentsply Sirona, Konstanz, Germany).

All sixty roots were randomly divided into 2 groups, based on the adopted resin cements: Rely X U200 (3M ESPE, St Paul, MN, USA) and MaxCem Elite (Kerr, Orange, CA, USA).

The cement was stored in refrigerator (between 4° and 6°C) and protected from exposure to light, based on manufacturer’s specifications, until the expiration dates of the investigated products had exceeded the times established for evaluation in the current study.

Each group was separated into 3 subgroups (n=10), based on the shelf life of each cement, namely: 1) Within the use time determined by the manufacturer; 2) 6 months after opening the aluminum blister; 3) 12 months after opening the aluminum blister.

After dividing the groups, dental canals were enlarged with the aid of Largo drill system 1, 2 and 3 (0.7, 0.9, and 1.1 mm in diameter, respectively), 4 mm away from the apical foramen. Then, the drill provided by the manufacturer, which corresponded to the fiberglass post diameter, was used at low speed.

All roots were subjected to conduit irrigation with 5 mL of distilled water and drying with absorbent paper cones (Meta Biomed, Tokyo, Japan). Exacto 1 pins (Angelus, Londrina, PR, Brazil) and gloves were cleaned with absolute alcohol; then, silane layer (Angelus) was applied to the pin. Double activation and self-adhesive resin cements ‘Rely X U200’ and ‘MaxCem Elite’ were manipulated based on using self-mixing tip, specific to each commercial brand. They were applied inside the canal, based on using the same tips, depending on the experimental group according different cements, as follows:

• Cemented with Rely X U200 (Rely X U200), which were subdivided in: Within the use time determined by the manufacturer or no expiration date, (NED; n=10); 6 months after opening the aluminum blister (indicated by the manufacturer as expiration date), (6 months; n=10); 12 months after opening the aluminum blister (indicated by the manufacturer as expiration date), (12 months; n=10).

• Cemented with MaxCem Elite (MaxCem Elite), which were subdivided in: Within the use time determined by the manufacturer or no expiration date, (NED; n=10); 6 months after opening the aluminum blister (indicated by the manufacturer as expiration date), (6 months; n=10); 12 months after opening the aluminum blister (indicated by the manufacturer as expiration date), (12 months; n=10).

Resin cement excess was removed based on using microbrush; all roots were photoactivated for 120 s, with LED photoactivator (Radii-cal; SDI, São Paulo, SP, Brazil) at irradiance of 1,400 mW/cm².

Roots were preserved in microorganism-free microbiological oven, at 37ºC and 100% humidity, for 24 hours. All roots were prepared and restored by a single trained operator, who followed manufacturers’ recommendations.

After 24 hours, roots were positioned, trapped in acrylic resin block and cross sectioned into slices, in order to produce 1mm specimens. This procedure was carried out in cutting machine ( Isomet 1000; Buehler Ltd, Lake Bluff, IL, USA) equipped with diamond steel disk (Extec 12205; Extec Corp. Enfield, CT, USA), operating at 200 rpm, under water conduction. The first cut was performed 1 mm away from the amelocementary limit, and hus successively, until 2 cuts per portion - cervical, middle and apical – were obtained, ( Table 1).

-Microhardness Test

Measurements were taken 24 hours after cementation for microhardness test purposes. Surface microhardness of all samples was measured based on using hardness testing machine (HMV-G; Shimadzu, Kyoto, Japan) equipped with Knoop-type indenter operating at- static load of 25 g applied every 5 s. Three indentations were performed on each sample in the elliptical cementing line with an angle of 120° between them. Knoop hardness number (KHN) was expressed as the mean of three indentations performed in the same sample (12).

-Bond Strength Test

Bond strength test was carried out by extruding the fiberglass pin. The specimen was positioned on a metal device - whose central opening was larger than the diameter of the canal- with the coronal portion of it facing down; 0.6 mm diameter cylinder tip connected to universal testing machine (EMIC, São José dos Pinhais, PR, Brazil) was used at the speed of 1.0 mm/min (11).

Adhesive strength (σ) was measured based on the following formula: σ = C/A

wherein: C = specimen rupture load (kgf) and A = interfacial area (mm²)

Specimens’ interfacial area (A) was calculated based on the formula used to calculate the lateral area of the trunk of a right circular cone with parallel bases.

A = π x G x (R1 + R2)

wherein: A = interfacial area, π = 3.14, G = change generator, R1= smallest base’s radius and R2 = largest base’s radius

Pythagorean theorem was used to calculate the generatrix of the truncated cone G: “the sum of the squares of the lengths of the triangle’s legs is the same as the square of the length of the triangle’s hypotenuse”. The following formula was used to calculate G: G2 = h2 + [R2 – R1]2

wherein: h = section height, R1 = smallest base’s radius and R2 = largest base’s radius

R1 and R2 values were obtained by measuring the internal diameters of the smallest and largest bases, respectively, which corresponded to the internal diameter between the walls of the specimen’s canal and then divided by 2 to obtain the radius. These diameters and specimen height were measured with digital caliper (Starret 727; Starret Itu, Itú, SP, Brazil), before the test.

Adhesive strength σ result was initially expressed in kgf/mm², which was transformed into MPa, by multiplying the σ value by 9.8, based on the following equation of measurements: 1kgf/ mm² = 9.8 N/ mm² = 9.8 MPa.

All fiber post specimens and all three root thirds were assessed in optical light microscope, at maximum magnification of 40x (BEL Equipamentos Analíticos Ltda., *Pi*racicaba, SP, Brazil). Fractures were classified in four categories, as follows:

1) Adhesive fracture between the fiber post and resin cement;

2) Adhesive fracture between the resin cement and root dentin;

3) Cohesive fracture of the fiber post;

4) Mixed fracture (when more than one of the highest classifications appear on the same specimen).

-Statistical analysis

Collected data were analyzed in Minitab 16 software for Windows 8 (Minitab, State College, PA, USA). They were subjected to Shapiro-Wilk normality test, which was followed by non-parametric (for hardness) and parametric (for bond strength) tests. Hardness data were subjected to Kruskal-Wallis test (Factors: Material, Time and Root thirds), whereas bond strength data were subjected to analysis of variance (Factors: Material, Time and Root thirds), followed by Tukey test; both analyses were performed at 5% significance level (α = 0.05).

## Results

**Figure 1:**
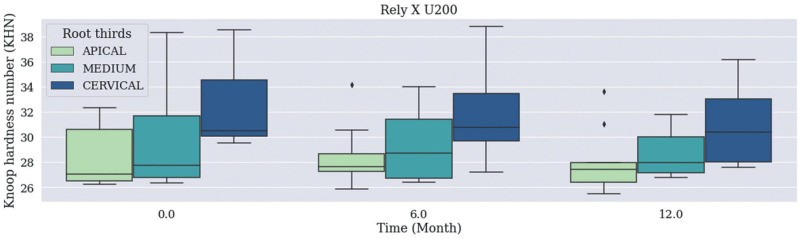
Median Knoop Hardness values recorded for Rely X U200 Cement based on the Material / Time / Root Thirds combination. Time was measured in months: 0 (no expiration date); 6 (6 months after expiration date), 12 (12 months – 1 year after expiration date).

**Figure 2:**
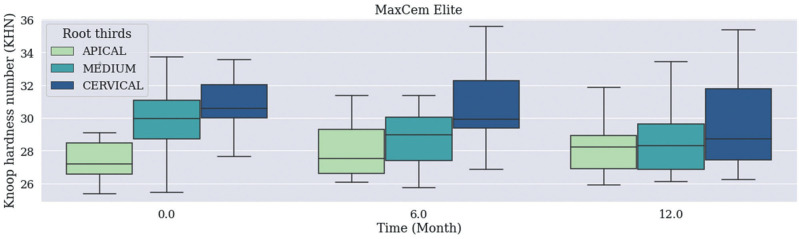
Median Knoop Hardness values recorded for MaxCem Elite Cement based on the Material / Time / Root Thirds combination. Time was measured in months: 0 (no expiration date); 6 (6 months after expiration date), 12 (12 months – 1 year after expiration date).

**Figure 3:**
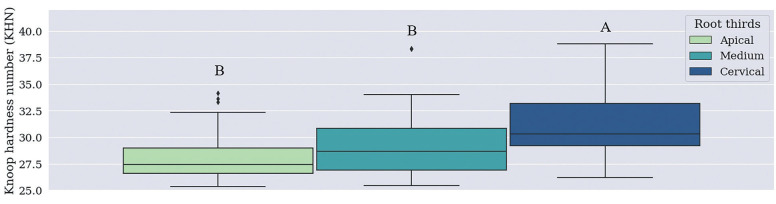
Median Knoop hardness number (KHN) values recorded for independent factor ‘Root thirds’. Different letters show statistically significant difference in independent factor ‘Root third’ (Kruskal-Wallis test).

There was no statistically significant difference in median Knoop Hardness values recorded for the Material / Time / Root Thirds combination (Figs. 1, 2), (*p*=0.483) and for independent Material and Time factors. Cervical third recorded higher Knoop Hardness values when the root third region was used as independent factor than when the other thirds, which did not differ from each other, were used for such a purpose (Fig. 3, *p*<0.001).

There was no statistically significant difference in mean values recorded for bond strength based on the Material / Time / Root Thirds combination (*p*=0.237). According to  Table 2 (*p*<0.001), Rely X U200 resin cement has recorded mean bond strength values for independent factor ‘Material’ significantly higher than the ones recorded for MaxCem Elite resin cement.

According to  Table 3 (*p*<0.001), shelf life expired after 12 months recorded mean bond strength values for independent factor ‘time’ significantly higher than the ones observed for shelf life expired after 6 months and for cements within the use-time recommended by the manufacturer, which did not differ from each other.

According to  Table 4 (*p*<0.001), cervical third recorded mean bond strength values for independent factor ‘root third’ significantly higher than the ones observed for the other thirds, which did not differ from each other.

**Figure 4:**
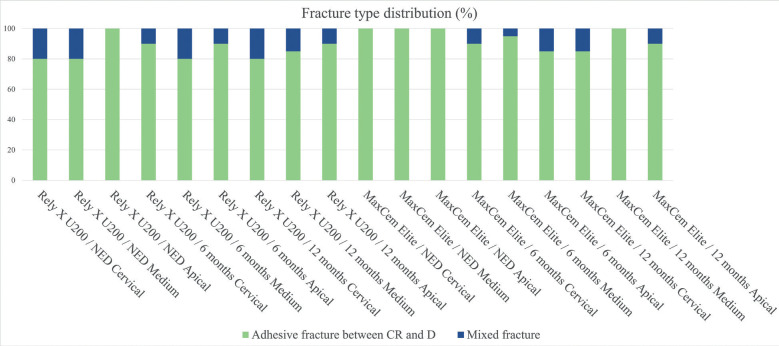
Fracture type distribution based on Material, Time and Root third (%). CR: composite resin; RC: resin cement; IR: intraradical retainer; D: dentin. NED: No expiration date. 6 months: 6 months after expiration date. 12 months: 12 months after expiration date. There was no fracture distribution for the following fracture types: adhesive fracture between fiber post and resin cement; and cohesive fiber post fracture.

According to Fig. 4, the adhesive fracture between cement and dentin was the most frequent fracture pattern; it was followed by mixed fracture.

## Discussion

The following features were assessed in the current study: bond strength between fiberglass post and root dentin treated with self-adhesive resin cements with expired shelf life (6 months and 1 year) and these cements’ hardness. Results (Figs. 1,2) have evidenced that shelf life-expired materials did not show statistically significant difference in median hardness values recorded for the Material / Time / Root Thirds combination, or statistically significant difference in mean bond strength values recorded for this very same combination (*p*=0.237).

Median microhardness values recorded for independent factors ‘material’ and ‘time’ did not show statistically significant differences between groups subjected to commercial brands ‘Rely X U200’ and ‘MaxCem Elitte’, among expiration dates (6 months, 12 months and within the use-time recommended by the manufacturer, respectively). This finding may be associated with the way the material was stored – i.e., in refrigerator, at low and controlled temperature, without exposure to light -, which delayed the degradation of components’ physical-chemical properties and kept their chemical stability, regardless of storage time, as previously reported in studies carried out with resinous composites (7,13).

Based on the Knoop hardness test applied to root thirds (Fig. 3), cervical third microhardness recorded higher values (30.3) than the ones observed for the middle (28.7) and apical thirds (27.4). This finding can be explained by the fact that the cervical region was closer to the light incidence focus, which is the curing light used to trigger the setting in a light-activated way, whereas the middle and apical thirds were further away from it (14,15-17). Because they received lower light incidence, their setting was mainly chemical, and it may have resulted in lower hardness after the final setting, in comparison to the cervical region. Studies carried out by Vignolo *et al*. (2012) (16) and Geng *et al*. (2020) (17) have found similar results, according to which, microhardness has gradually decreased along the root canal.

Mean bond strength values ( Table 2) recorded for Rely X U200 resin cement (30.3) were significantly higher than the ones observed for MaxCem Elite resin cement (28.7). Despite the fact that most resin cements have similar overall chemical composition, as well as ionized multifunctional methacrylate monomers capable of reacting to the hydroxyapatite mineral portion of the tooth structure to promote adhesion (4), companies accounting for each chemical brand implemented some changes to improve their product. Chemical composition, viscosity and pH stand out among factors capable of influencing self-adhesive cements’ interaction with the substrate (12,14,18). MaxCem Elite has maintained low pH (2.2), whereas U200 pH has increased from 2.8 to 7.0, 24 h after cementation. Some authors suggest that keeping the pH low may have adverse effect on bond strength between self-adhesive cements and root dentin (12,14,18) - this effect was observed in results in the study.

With respect to mean bond strength values recorded based on independent factor ‘time’ ( Table 3), 1-year-expired shelf life, recorded significantly higher mean values (20.9) than the ones observed for 6-month-expired shelf life (16.1) and for products within the use-time recommended by the manufacturer (17.6), which did not differ from each other. This feature can be explained by the preservation of products’ chemical composition, due to time and storage conditions (7,13). However, little is known about the physicochemical features of self-adhesive resin cements after expiration date due to lack of research in the literature about this topic. Thus, further studies should be conducted in this field to help improving knowledge about composite resins and their properties. In addition to being a limitation of the present study.

The highest mean bond strength value recorded for root region ( Table 4) was observed in the cervical third, which presented significantly higher mean values (20.3) than the ones observed for the middle (17.3) and apical thirds (17.1), which did not differ from each other. It happened due to the prevalent polymerization type observed in this region, which was similar to the one mentioned in microhardness (14,16,17,19). It also happened due to the larger number and permeability of dentinal tubules in the cervical portion of the root, than those observed for the medium and apical thirds. This finding is in compliance with results reported in similar studies (15,20).

Adhesive fracture between cement and dentin (Fig. 4) was the fracture pattern most often observed in the current study. This finding goes against results in studies previously carried out in this field, which have concluded that the cement-dentin interface was the site showing the highest sensitivity and stress concentration (15,21-25).

Based on results in the present study, it was possible seeing that the shelf life (expired after 6 months and 1 year) of self-adhesive resin cements kept under the ideal conditions recommended by the manufacturer did not influence material’s microhardness and bond strength. It is important pointing out that, according to SESA Resolution n. 496/2005, dentists are the ones accounting for monitoring the shelf life of all dental materials used by them. Failing to comply with this duty is a violation that leads to the application of fines to be paid by these professionals, not to mention that this practice can hinder the treatment provided to patients.

The current study does not intend to validate the use of products with expired shelf life, since it only assessed two properties of the investigated cements. Thus, future studies about this topic should be conducted before one can suggest that the shelf life of these products should be expanded. Thus, based on results in the present study, it was possible concluding that shelf life expired after 6 months and 12 months did not affect the microhardness and bond strength of self-adhesive resin cements stored under the ideal conditions recommended by the manufacturer.

## Figures and Tables

**Table 1:** Resin cements’ description.

Product	Presentation	Working time / Shade	Composition
MaxCem Elite (KERR) - Kerr, Orange, CA, EUA	Syringe ( base + catalyst); Direct dispenser through self-mixing tip	2 min + 3 min gel time (based on oral temperature) / Yellow	GPDM, comonomers (mono-, di-, and tri-functional methacrylates); self-curing redox activator, photoinitiator (camphorquinone), stabilizer, fluoroaluminosilicate glass particles, silica.
Relyx U200 (3M ESPE) - 3M ESPE, St Paul, MN, EUA	Syringe ( base + catalyst); Direct dispenser through self-mixing tip	2 min – 5 min at 22ºC / A2 Universal	Base paste: silane-treated glass powder, 2-propenoic acid, 2-methyl, 1,'-[hydroxymethyl)-1,2-ethanediyl] ester, triethylene glycol dimethacrylate (TEG-DMA), silane-treated silica, fiberglass, sodium persulfate and t-butyl per-3,5,5-trimethylhexanoate. Catalyst paste: silane-treated glass powder, substituted dimethacrylate, silane-treated silica, sodium p-toluene sulfate, 1-benzyl-5-phenylbaric acid, calcium salts, 1,12-dodecane dimethacrylate, calcium hydroxide and titanium dioxide.

**Table 2:** Mean Bond Strength (MPa) values recorded for independent factor ‘Material’.

Material	Bond Strength (MPa)
Rely X U200	30.3 (3.6) A
MaxCem Elite	28.7 (3.1) B

Different letters show statistically significant difference in independent factor ‘Material’ (p<0.001; Tukey’s test). Standard deviations are shown in parentheses.

**Table 3:** Mean Bond Strength (MPa) values recorded for independent factor ‘Time’.

Time (months)	Bond Strength (MPa)
12 months	20.9 (3.1) A
6 months	16.1 (4.4) B
NED	17.6 (3.6) B

Different letters show statistically significant difference in independent factor ‘Time’ (p<0.001; Tukey test). Standard deviations are shown in parentheses. 12 months: 12 months after expiration date. 6 months: 6 months after expiration date. NED: No expiration date.

**Table 4:** Mean Bond Strength (MPa) values recorded for independent factor ‘Root thirds’.

Root thirds	Bond Strength (MPa)
Cervical	20.3 (5.1) A
Medium	17.3 (3.8) B
Apical	17.1 (3.1) B

Different letters show statistically significant difference in independent factor ‘time’ (p<0.001; Tukey test). Standard deviations are shown in parentheses.

## Data Availability

The datasets used and/or analyzed during the current study are available from the corresponding author.
